# Fibroblast Growth Factor-2 Enhanced The Recruitment of Progenitor Cells and Myelin Repair in Experimental Demyelination of Rat Hippocampal Formations 

**DOI:** 10.22074/cellj.2015.14

**Published:** 2015-10-07

**Authors:** Mahdieh Azin, Javad Mirnajafi-Zadeh, Mohammad Javan

**Affiliations:** Department of Physiology, Faculty of Medical Sciences, Tarbiat Modares University, Tehran, Iran

**Keywords:** FGF2, Hippocampus, Neural Stem Cells

## Abstract

**Objective:**

Hippocampal insults have been observed in multiple sclerosis (MS) patients. Fibroblast growth factor-2 (FGF2) induces neurogenesis in the hippocampus and en- hances the proliferation, migration and differentiation of oligodendrocyte progenitor cells (OPCs). In the current study, we have investigated the effect of FGF2 on the processes of gliotoxin induced demyelination and subsequent remyelination in the hippocampus.

**Materials and Methods:**

In this experimental study adult male Sprague-Dawley rats re- ceived either saline or lysolecithin (LPC) injections to the right hippocampi. Animals re- ceived intraperitoneal (i.p.) injections of FGF2 (5 ng/g) on days 0, 5, 12 and 26 post-LPC. Expressions of myelin basic protein (*Mbp*) as a marker of myelination, *Olig2* as a marker of OPC proliferation, *Nestin* as a marker of neural progenitor cells, and glial fibrillary acidic protein (*Gfap*) as a marker of reactive astrocytes were investigated in the right hippocampi by reverse transcriptase-polymerase chain reaction (RT-PCR).

**Results:**

There was reduced *Mbp* expression at seven days after LPC injection, in- creased expressions of *Olig2* and *Nestin*, and the level of *Gfap* did not change. FGF2 treatment reversed the expression level of *Mbp* to the control, significantly enhanced the levels of *Olig2* and *Nestin*, but did not change the level of *Gfap*. At day-28 post- LPC, the expression level of *Mbp* was higher than the control in LPC-treated animals that received FGF2. The levels of *Olig2*, *Nestin* and *Gfap* were at the control level in the non-treated LPC group but significantly higher in the FGF2-treated LPC group.

**Conclusion:**

FGF2 enhanced hippocampal myelination and potentiated the recruitment of OPCs and neural stem cells (NSCs) to the lesion area. Long-term application of FGF2 might also enhance astrogliosis in the lesion site.

## Introduction

The hippocampus, which has an essential role in learning and memory, is sensitive to different insults including the demyelination that occurs as a result of multiple sclerosis (MS) (1). The extension of demyelination into the hippocampal formation seems to be responsible for some cognitive disorders such as long-term memory impairment reported in patients who suffer from MS (2). The myelinated fibers of the perforant path are the main afferent source of the hippocampus and apparently hippocampal demyelination can affect their performance. The hippocampus contains neural stem cells (NSCs) in its sub-granular zone which are able to differentiate into neurons and glial cells; therefore it is an appropriate location for studying demyelination and the subsequent endogenous remyelination process (3). Direct injection of demyelinating gliotoxins into the white matter leads to demyelination and the consequent remyelination process (4,5). Our previous study has shown that gliotoxins could induce distinct demyelination and remyelination phases in the hippocampus as determined by histological, electrophysiological and molecular analyses (6). Therefore gliotoxin induced-hippocampal demyelination provides an adequate experimental model for evaluating the protective effect of different potential therapies on the myelinating cells or for evaluating their effects on the efficacy of remyelinating mechanisms, especially when the new therapies are expected to target the cognitive aspects of MS. 

Multipotent NSCs, as the functional reservoir for brain tissue repair, renew themselves and differentiate to new neurons or glial cells according to the special phenotype that the niche dictates (7,8). Appropriate environmental signals such as growth factors and chemicals that modify signal transduction pathways can contribute to efficient proliferation, migration and final differentiation of NSCs (9). Growth factors brain-derived neurotrophic factor (BDNF), fibroblast growth factor-2 (FGF2) and epidermal growth factor (EGF) are known as the mediators of adult neurogenesis (10). FGF2 regulates the responses of the oligodendrocyte lineage, which include proliferation and migration, followed by final differentiation of precursors into oligodendrocytes (11). *In vivo* and *in vitro* studies have suggested different effects of FGF2 on remyelination based on the administration pattern of FGF2. Continuous FGF2 administration (twice daily for 3 days) has been shown to cause loss of myelin-forming oligodendrocytes and myelin in the adult central nervous system (CNS) while transient exposure to FGF2 (single dose) increased myelination in an *in vivo* study (12,13). When demyelination was induced in the optic chiasm of mice, FGF2 could potentiate remyelination (14). 

Myelin basic protein (*Mbp*), a component of myelin proteins essential for myelin compaction and stability (15), is frequently used as an index of myelination (14,17). The decreased *Mbp* expression reflects the reduced number of myelinating oligodendrocytes. Changes in the expression of *Olig2* show the relative number of activated oligodendrocyte progenitor cells (OPCs) at the site of demyelination (18). In several studies, *Nestin* (intermediate filament protein VI) is used as a biological marker to determine NSCs (19). Glial fibrillary acidic protein (*Gfap*) is expressed by reactive astrocytes at the lesion sites. Although both harmful and beneficial activities have been attributed to these cells, recent studies show roles for reactive astrocytes in limiting inflammation and protecting neurons and oligodendrocytes in lesions (20). 

The current study investigated the effects of FGF2 on the responses of myelinating cells, OPCs and reactive astrocytes in rat hippocampi following local injections of lysolecithin (LPC) at days 7 and 28 post-demyelination induction. 

## Materials and Methods

### Animals

For this experimental study 24 adult male Sprague-Dawley rats were obtained from Razi Institute, Karaj, Iran. Rats weighed 280-320 g and were maintained 4 per cage under a 12 hour light/ dark cycle in a room with controlled temperature (24 ± 2˚C). Food and water were available ad-libitum. All research and animal care procedures were performed according to International Guidelines on the Use of Laboratory Animals and were approved by the Ethical Committee for Animal Research at Tarbiat Modares University. 

### Surgery procedure and treatment

Animals received saline (control group) or LPC to induce hippocampal demyelination. For induction of demyelination, rats were anaesthetized with intraperitoneal (i.p.) injections of combined ketamine (10 mg/kg) and xylazine (2 mg/kg) after which they each received a 1.5 µl injection of 1% LPC dissolved in sterile normal saline. Injections were performed using a 30-gauge needle attached to a 5-µL Hamilton syringe into the right hippocampus (coordinates: A, 5 mm; L-3 mm; V, 2.5 mm below the dura). Animals were divided into three groups: control, non-treated LPC, and FGF2treated LPC. Animals in the FGF2-treated group received i.p. injections of 5 ng/g FGF2 (Royan, Iran) (21) once/day before the injection of LPC and on days 5, 12 and 26 post-LPC injection. 

### Gene expression study

Based on our previous studies in rats (6,14), we have determined that demyelination is the most active process within the first week and can be FGF2 and Hipocampal Demyelinat ion observed at day 7. Therefore day 7 is considered adequate for assessing the effect of treatments on demyelination. Up to day 28 (week 4), the major phenomenon is remyelination; therefore day 28 appears to be adequate for assessing the effect of treatments on the remyelination process. 

For the gene expression study, rats were killed while under CO _2_-induced light anesthesia by rapid decapitation at days 7 or 28 post-LPC injection; the right hippocampi were removed and immediately frozen in liquid nitrogen (n=6-8 for each group/ data point). For dissection of the hippocampus, the rat brain was cut along the longitudinal fissure by a surgical blade and then the regions posterior to the lambda were removed. The cerebral hemisphere was then placed medial side up for removing the diencephalon and exposing the medial side of the hippocampus. The final dissection of tissue was performed with a needle-tip. 

We measured expressions of the *Mbp*, *Olig2*, *Nestin* and *Gfap* genes by semi-quantitative reverse transcriptase-polymerase chain reaction (RT-PCR) as previously described (22). Primer sequences used for the amplification of *Mbp*, *Olig2*, *Nestin* and *Gfap* cDNA were designed on the basis of sequences in the Gene Bank. Primer sequences, the length of PCR products, amplification cycles, annealing temperature, and the amount of cDNA template used for each reaction are presented in table 1. The PCR products were resolved on agarose gel, visualized and photographed using a gel documentation apparatus, and finally quantified with band densitometry. 

### Statistical analysis

Results are expressed as mean ± standard error of mean (SEM). For gene expression analysis, the values of the band density were measured using band densitometry by Image J software. Values were subsequently normalized to *Gapdh* band density at the same sample. Then, the normalized values were averaged for each group and compared statistically. Statistical assessments were performed using one-way analysis of variance followed by LSD post-hoc. We considered P values <0.05 to be statistically significant.

## Results

### Effect of fibroblast growth factor-2 on Mbp expression

**Fig.1:**
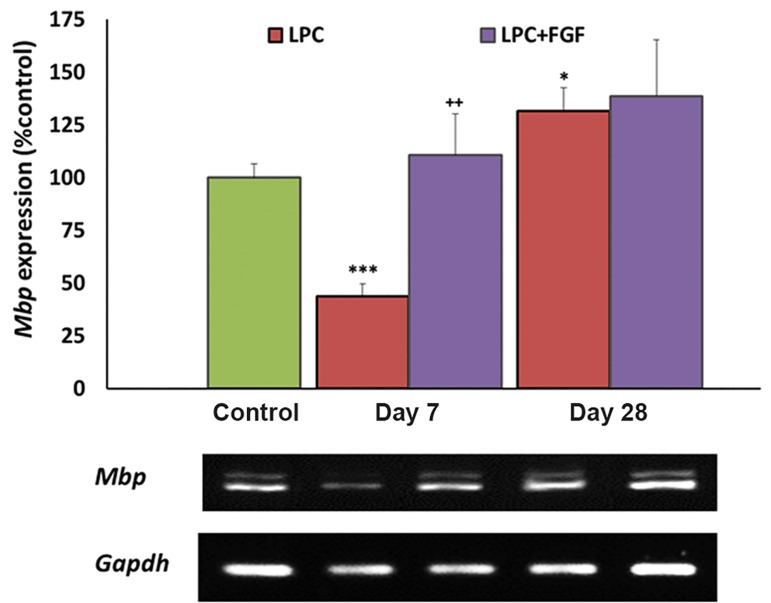
Evaluation of the effect of fibroblast growth factor-2 (FGF2) on myelin basic protein (Mbp) gene expression by semi-quantitative reverse transcriptase-polymerase chain reaction (RT-PCR) following local injection of lysolecithin (LPC). We observed increased Mbp gene expression at day 7 in the FGF2-treated group. Bars: Mean ± standard error of mean (SEM), n≥5, *; P<0.05,***; P<0.001 compared to the control group and ++; P<0.01 compared to the non-treated group at day 7 as evaluated by the LSD test.

The expression level of *Mbp* was measured as an index of myelination by myelinating oligodendrocytes. At seven days after LPC injection, there was a significant decrease in the level of *Mbp* (P<0.001). Although in the FGF2-treated group there was no significant change in the expression of Mbp when compared to the control, however the expression was significantly higher than the LPCtreated group. At day 28, there was no significant difference between FGF2-treated and non-treated groups, while the expression level was more than the control group (P<0.05, Fig .1).

**Table 1:** Primer sequences and characteristics of the polymerase chain reaction (PCR) reactions used for amplifications of myelin basic protein *(Mbp), Olig2, Nestin* and glial fibrillary acidic protein *(Gfap)* cDNAs


Genes	Primer sequence	Product size (kb)	Number of cycles	T annealing (˚C)	cDNA (μl)

*Mbp*	F: CACAGAAGAGACCCTCACAG	337/415	26	56	2
	R: CCCCAGCTAAATCTGCTGAG				
*Olig2*	F: ACACCACCACGTGTCGGCTA	450	30	56	3
	R: CTGCGTCTCTTCTAAGCCCAGA				
*Nestin*	F: GGAGTGTCGCTTAGAGGT	483	30	58.4	2
	R: GGAGTGTCGCTTAGAGGT				
*Gfap*	F: CTCGTGTGGATCTGGAGAGGAA	572	26	56	2
	R: GCCCTCCAGCAATTTCCTGTAG				
*Gapdh*	F: AAACCCATCACCATCTTCCA	198	24	56	1
	R: GTGGTTCACACCCATCACAA				


### Effect of fibroblast growth factor-2 on Olig2 expression

**Fig.2:**
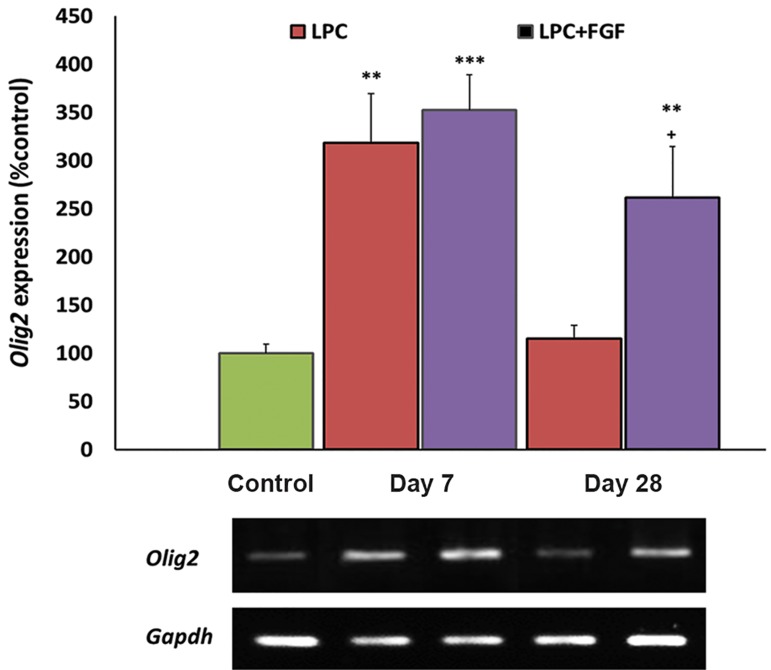
Evaluation of the effect of fibroblast growth factor-2 (FGF2) on Olig2 gene expression by semi-quantitative reverse transcriptase-polymerase chain reaction (RT-PCR) following local injection of lysolecithin (LPC). FGF2 increased the expression of Olig2 at day 28 compared to the non-treated group. Bars: Mean ± standard error of mean (SEM), n≥5, **; P<0.01, ***; P<0.001 compared to the control group and +; P<0.051 compared to the non-treated group at day 28 as evaluated by the LSD test.

*Olig2* expression level was measured as an index of the presence oligodendrocyte precursors. Following local injection of LPC into the hippocampus, we observed a significantly increased expression level of *Olig2* at day 7 (P<0.01) that returned to the control level at day 28 post-LPC injection. When the LPC injected animals were treated with FGF2, the expression levels of *Olig2* significantly increased at both 7 (P<0.001) and 28 days (P<0.01) post-LPC. When compared with non-treated animals, FGF2 treated animals showed higher levels of *Olig2* expression at day 28 post-LPC (P<0.05, Fig .2).

### Effect of fibroblast growth factor-2 on Nestin expression

**Fig.3:**
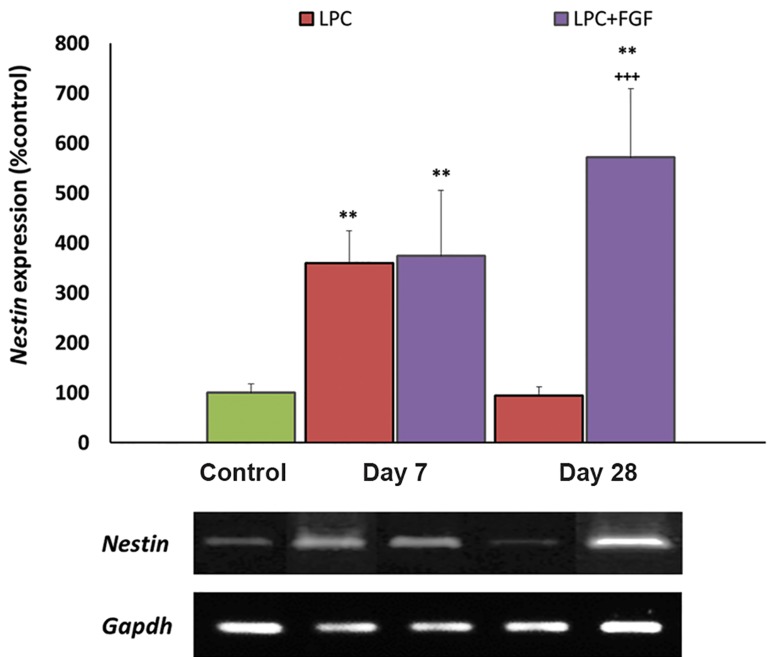
Evaluation of the effect of fibroblast growth factor-2 (FGF2) on Nestin gene expression by semi-quantitative reverse transcriptase-polymerase chain reaction (RT-PCR) following local injection of lysolecithin (LPC). An increased expression of Nestin was observed at day 28 in the FGF2-treated group. Bars: Mean ± standard error of mean (SEM), n≥5, **; P<0.01 compared to the control group and +++; P<0.001 compared to the non-treated group at day 28 as evaluated by the LSD test.

*Nestin* gene expression was measured to monitor the recruitment of NSCs within the hippocampal lesions. Both FGF2-treated and non-treated groups showed increased expressions of *Nestin* within the lesion area at 7 days post-LPC injection (both P<0.05). At 28 days post-LPC, the expression level of *Nestin* returned to the control level in the non-treated group, but it remained higher than the control group in FGF2-treated animals. At day 28, the gene expression level of *Nestin* in the FGF2-treated group was significantly higher than the non-treated group (P<0.001, Fig .3).

### Effect of fibroblast growth factor-2 on *Gfap* expression

**Fig.4:**
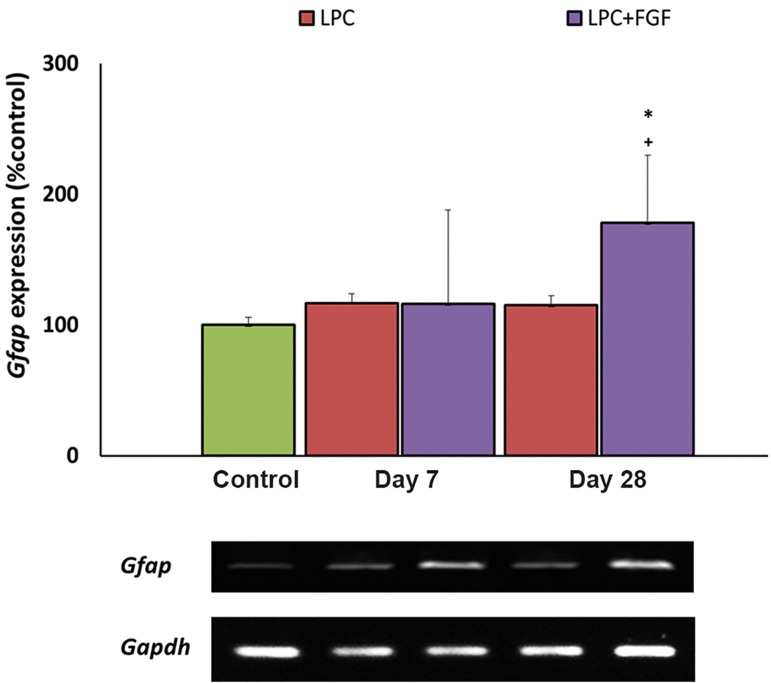
Evaluation of the effect of fibroblast growth factor-2 (FGF2) on the expression level of glial fibrillary acidic protein (Gfap) by semi-quantitative reverse transcriptase-polymerase chain reaction (RT-PCR) following local injection of lysolecithin (LPC). An increased expression of Gfap was observed at day 28 in the FGF2- treated group. Bars: mean ± standard error of mean (SEM), n≥5, *; P<0.01 compared to the control group and +; P<0.05 compared to the non-treated group at day 28 as evaluated by the LSD test.

We monitored the *Gfap* expression level in order to measure for a possible effect of FGF2 on astrocyte reactivation within the lesion area *Gfap*. At 7 days post-LPC injection, none of the treated and non-treated groups of animals showed changes in the level of *Gfap*, while at 28 days post-lesion, the expression level of *Gfap* was significantly higher in the FGF2-treated group when compared with the control and nontreated animals (both P<0.05, Fig .4).

## Discussion

this study, we measured the extent of LPC-induced local demyelination of the hippocampus by monitoring the expression level of *Mbp*. A decreased level of *Mbp* at day 7 and its reversal to a level higher than the control at day 28 confirmed the occurrence of previously reported demyelination and consequent remyelination by endogenous progenitors (22). The higher level of *Mbp* expression at day 28 was likely due to a higher level of *Mbp* expression by newly generated remyelinating cells in the lesion area. FGF2 increased the level of *Mbp* at day 7 post-LPC injection. This observation might be due to the protection of oligodendrocytes against LPC-induced lesions or accelerated myelin repair via endogenous progenitors (OPCs and NSCs). While both possibilities were plausible, the increased expressions of *Olig2* and *Nestin* implied that the increased myelin repair was at least partially responsible for higher myelination at day 7 in FGF2-treated animals. Based on a previous report, treatment with FGF2 in animals suffering from experimental autoimmune encephalomyelitis (EAE) reduced the extent of demyelination (23). Another study showed a dual effect of FGF2 on oligodendrocytes in the adult CNS. On the one hand FGF2 caused the destruction of adult oligodendrocytes and loss of myelin, but on the other hand it caused production of new immature oligodendrocyte cells (24). These reports also supported the possibility of increased myelin repair by FGF2 administration, as it was detected by increased *Mbp* expression at day 7. 

OPCs and immature oligodendrocytes express *Olig2* (25), therefore the recruitment of OPCs into the lesion area increases the level of *Olig2*. At day 7 post-LPC injection both FGF2-treated and nontreated groups were shown to have higher levels of *Olig2* which fortified the migration of OPCs into the lesion area. This finding concurred with a previous report concerning the migration of endogenous OPCs into demyelinated lesions in adult brains (26). While at day 28, the number of OPCs appeared to return to control levels in non-treated animals, this was mainly due to the differentiation of OPCs to myelinating cells; in FGF2-treated rats an increased number of OPCs in the lesion area was observed. This observation might be attributed to more extensive recruitment of OPCs by administration of FGF2. The same response was observed for *Nestin* expressing cells. NSCs appeared to migrate toward the lesion area at day 7 and differentiate to myelinating cells at day 28. Again FGF2 increased *Nestin* expression at day 28 which might imply more extensive recruitment of NSCs in FGF2-treated animals. This finding was supported by previous data in the demyelinated optic nerve where the treatment with FGF2 increased myelin repair and progenitor cell migration (14). Another study also showed that *Olig2* gene expression increased in response to FGF2 (27). Since FGF2 has been shown to increase the recruitment of NSCs and OPCs into the lesion area, accelerated myelin repair and the production of myelinating cells from progenitor cells might explain this discrepancy. As it was detected by higher expression of *Mbp* at day 7, it appeared that some progenitors differentiated into myelinating cells in the FGF2-treated group. Hence, we might consider higher total numbers of differentiated and non-differentiated cells in the FGF2-treated group at day 7. 

A previous study showed that a single injection of FGF2 could increase proliferation, recruitment, and migration of subventricular zone (SVZ) NSCs to a demyelinated area of the corpus callosum (21). Interestingly, in other experiments, continuous administration of FGF2 (twice a day, for 3 days) caused loss of myelin-forming oligodendrocytes and myelin loss in the adult CNS, whereas single administration of FGF2 increased myelination in an *in vivo* study (12,13). In the current study we injected FGF2 once per day on days 0, 5, 12 and 26. Although we repeated the injection of FGF2, this administration was non-continuous and we expected myelin repair by the formation of new myelinating oligodendrocytes. As a result, it seemed that non-continuous FGF2 administration caused progenitor cell proliferation and migration, after which these progenitors differentiated into myelinating cells. 

*Gfap* expression represents reactivated astrocytes at the lesion site. FGF2 administration had no effect on the level of *Gfap* expression at day 7 but it increased *Gfap* levels at day 28. Since both harmful and beneficial roles have been reported for the reactive astrocytes (20), more studies are required to elucidate the actual effect of *Gfap* expressing cells on myelin repair. Increased expression of *Gfap* at day 28 suggested that we should be concerned about its long-term administration. 

## Conclusion

Administration of FGF2 during demyelination and remyelination phases of LPC can potentiate myelination, probably by promoting the proliferation of NSCs and OPCs, and their migration toward demyelinating lesions.
